# Yellow nail syndrome linked to a mediastinal lipoma: a case report

**DOI:** 10.1186/s13256-024-04962-w

**Published:** 2024-12-11

**Authors:** Christoph Müller, Ingo Stricker, Peter Hykel, Dominic Dellweg

**Affiliations:** 1 Research, Philipps University of Marburg, Baldinger Straße, 35037 Marburg, Germany; 2 Institute of Pathology, Ruhr University Bochum, Bochum, Germany; 3 Department of Pulmonology, Fachkrankenhaus Kloter Grafschaft, Schmallenberg, Germany; 4 Department of Pulmonology, Pius-Hospital Carl Von Ossietzky University, Oldenburg, Germany

**Keywords:** Yellow nail syndrome, Chylothorax, Thoracic duct, Lymphatic drainage

## Abstract

**Background:**

Yellow nail syndrome is characterized by a yellow discoloration of the nails, respiratory symptoms, and lymphedema. It was first described in 1964 and has an estimated prevalence of less than 1:1.000.000. Despite its diverse manifestations affecting different organ systems and a wide range of associated diseases, yellow nail syndrome is most commonly related to impaired lymphatic drainage. The treatment depends on whether the underlying pathology can be identified and includes dietary, pharmacological, interventional, and surgical approaches.

**Case presentation:**

We report the case of a 73-year-old Caucasian male patient presenting with exertional shortness of breath and orthopnea, nonpitting edema of his distal extremities, and yellow discoloration of both his finger and toe nails. The diagnostic workup, which included the drainage of a large chylous pleural effusion, computed tomography of the chest, and lymphangiography, led to the diagnosis of yellow nail syndrome, presumably caused by a mediastinal lipoma compressing the thoracic duct. Treatment-wise, a percutaneous lymphatic embolization was performed after conservative treatment did not lead to a significant improvement of symptoms.

**Conclusion:**

While demonstrating the specific diagnostic findings of this case, we try to point out common pathogenetic aspects of the disorder and present the currently available treatment options.

## Background

Yellow nail syndrome (YNS) was first described by Samman and White in 1964 and is characterized by the clinical triad of yellow dystrophic nails, peripheral lymphedema, and respiratory symptoms [1]. It is categorized as a rare disorder with an estimated prevalence of less than 1:1.000.000 [2]. In general, it affects patients after the age of 50 years [3], although congenital forms are reported [4]. Most attempts to explain the pathophysiology of YNS involve an impaired lymphatic drainage. This can be owing to either an increased production of chyle or an obstruction of the thoracic duct, which drains the lymphatic fluid of the lower extremities and the chyle of the small intestines. While the presence of yellow nails is required to make the diagnosis, only one additional symptom of either lymphedema or a respiratory disorder is necessary [5]. In this case report, we present a patient with all three clinical features of YNS who underwent percutaneous lymphatic embolization after conservative treatment had failed. To our knowledge, this is the first reported case of YNS related to a benign mediastinal tumor.

## Case presentation

**Fig. 1:**
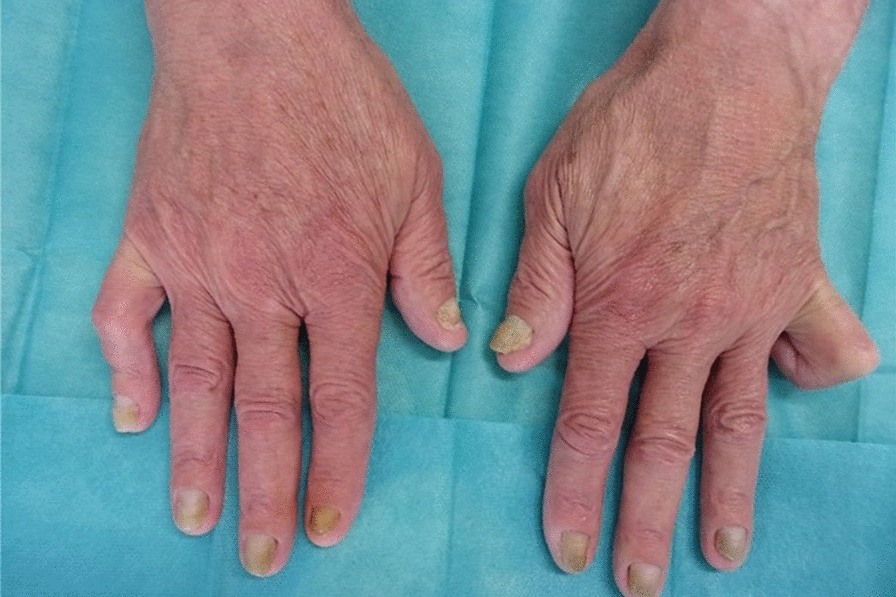
Fingernails—yellow discoloration and loss of the cuticles

**Fig. 2:**
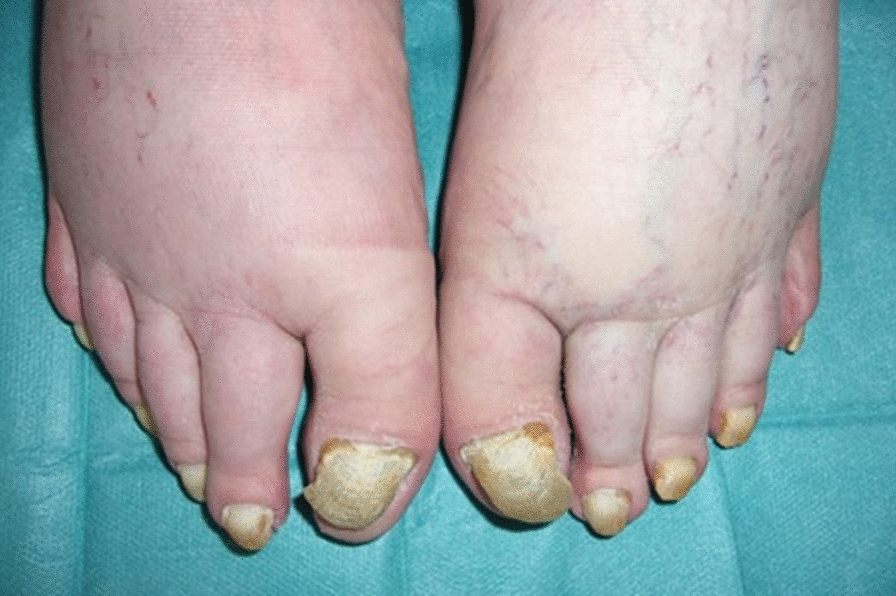
Toenails—yellow discoloration, thickening, and onycholysis

On his first admission, the 73-year-old Caucasian male patient was referred to our clinic due to new-onset dyspnea on exertion and orthopnea. He reported symmetrical swellings of the lower extremities for about 3 years and yellow discoloration of the finger and toenails for 2 years. The physical examination was notable for reduced breath sounds of the lower lung fields with dullness to percussion, peripheral edema including both feet and toes and yellow discoloration of the nails (Figs. 1, 2). The electrocardiogram was unremarkable except for a low voltage amplitude. On echocardiography, we observed a thin circumferential pericardial effusion of 6 mm during diastole without hemodynamic significance.

**Fig. 3:**
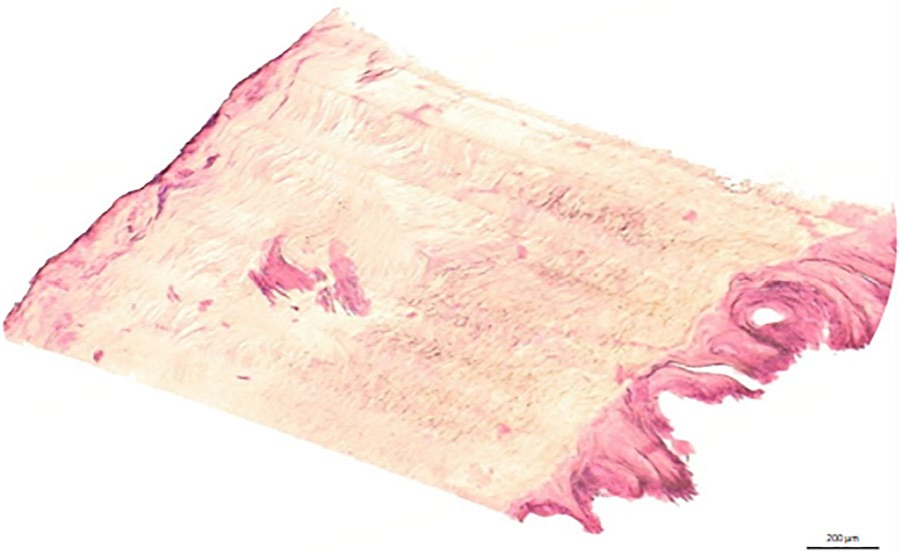
Thickened nail plate above and subungual stroma on the right side on hematoxylin and eosin staining

Pulmonary function testing showed an impaired forced exspiratory volume (FEV_1_) of 1.6 l (51.2%) and a reduced forced vital capacity (FVC) of 1.6 l (69.5%) with preserved ratio. The medical record of our patient included chronic bronchitis, epilepsy, one-sided amaurosis, hypothyroidism, resection of a retrobulbar meningeoma, and repeated surgical interventions of the lumbar spine. Our patient was on medical treatment with levetiracetam, l-thyroxin, valsartan, cholecalciferol, and tiotropium/olodaterol inhaler. The laboratory assessment of routine parameters was unremarkable. Nail biopsies were negative for onychomycosis on periodic acid–Schiff stain (PAS) and demonstrated nail thickening with dense subungual stroma on H&E staining (Fig. 3).

**Fig. 4:**
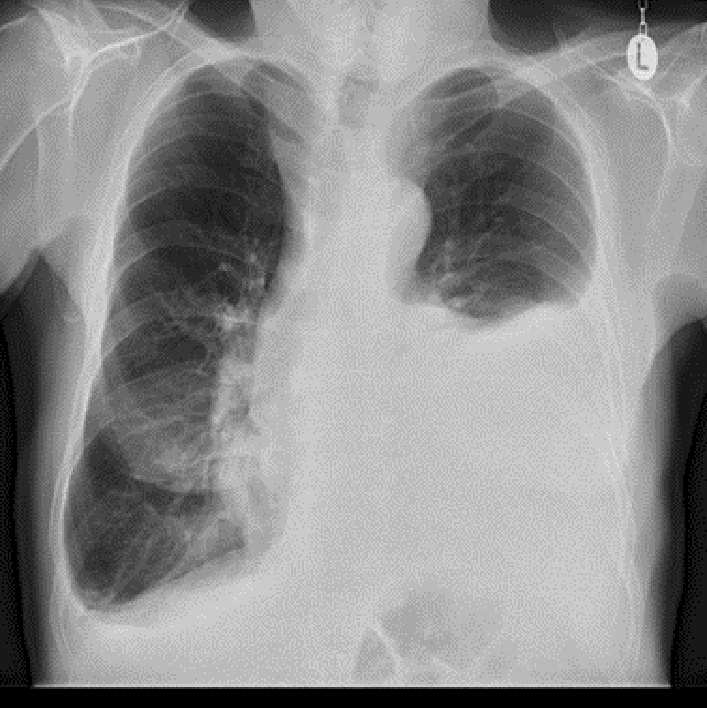
Chest X-ray—posteroanterior view

**Fig. 5:**
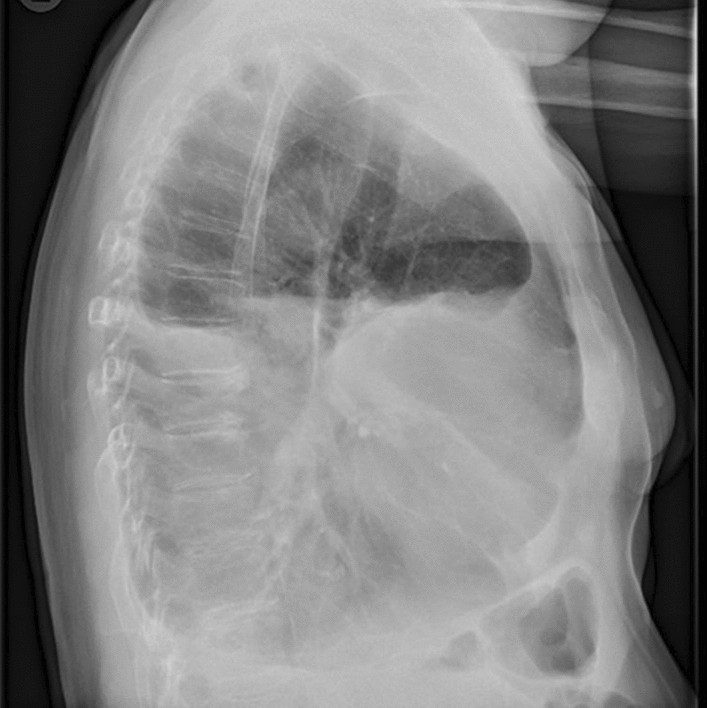
Chest X-ray—lateral view

**Fig. 6:**
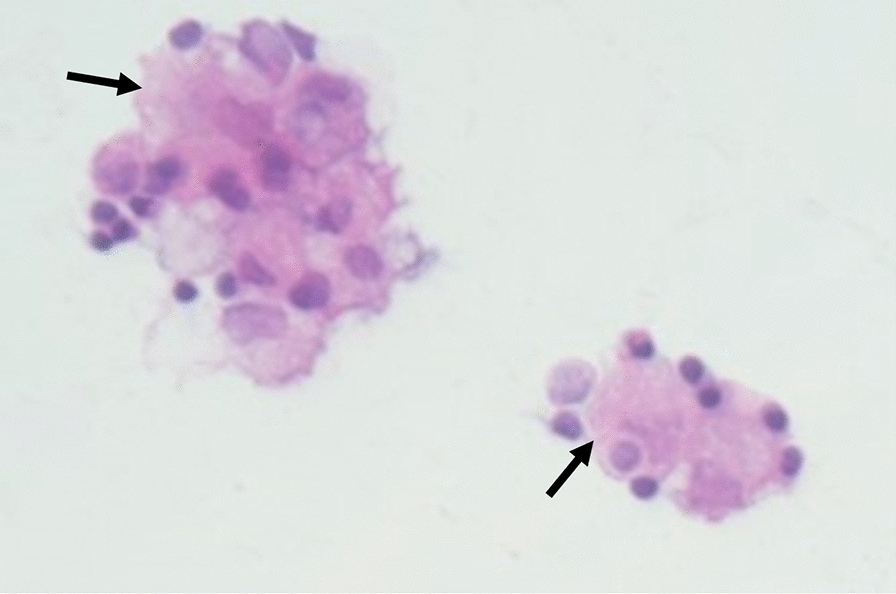
Foam cells indicated by arrows on hematoxylin and eosin staining

**Fig. 7:**
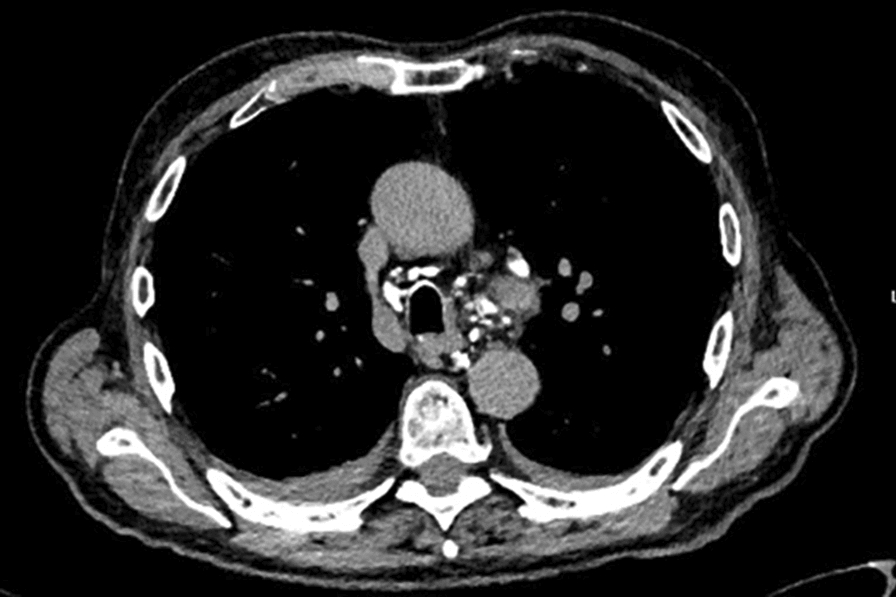
CT lymphangiography—transversal view, caudal to the mediastinal lipoma

**Fig. 8:**
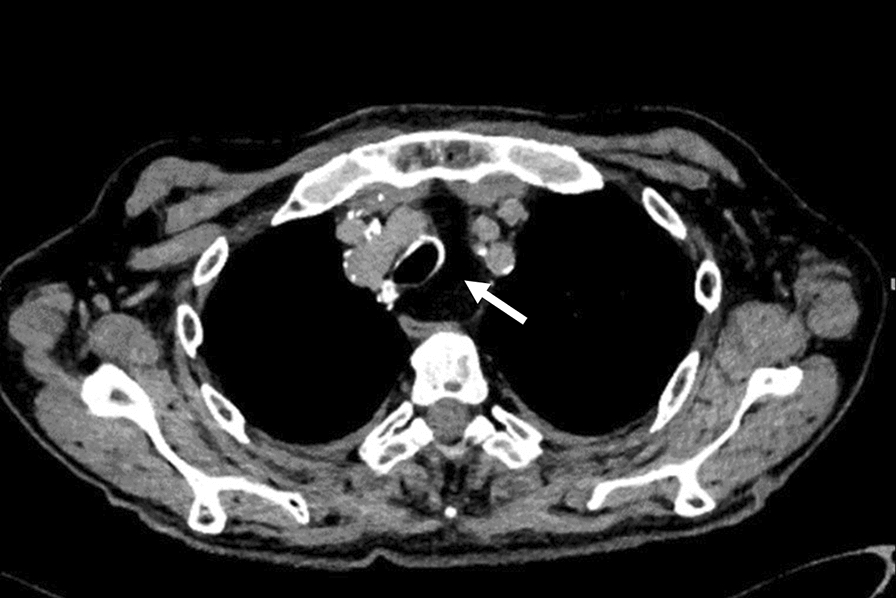
CT lymphangiography—transversal view, at the level of the mediastinal lipoma, as indicated by the white arrow

On chest X-ray, we observed a large pleural effusion of the left hemithorax and a meniscus sign on both sides (Figs. 4, 5). A left-sided ultrasound-guided puncture leading to the drainage of a chylous nonviscous fluid was performed. A draining catheter was placed subscapulary inside the ninth intercostal space. Laboratory assessment of the pleural fluid (Table 1) showed elevated concentrations of triglycerides and cholesterol with a triglyceride to cholesterol ratio of 1.3, confirming the suspected diagnosis of a chylothorax. Concentrations of protein and lactate dehydrogenase (LDH) were characteristic for an exsudate according to Light’s criteria. Cytopathological investigation showed foam cells on H&E staining (Fig. 6) and was positive for fat-specific staining with Sudan Red. Computed tomography (CT) of the chest revealed a 7 × 4.5 × 3 cm^3^ paratracheal lipoma without infiltrative growth or signs of compression of the surrounding mediastinal structures. At first, we decided for a dietary treatment approach and initiated total parenteral nutrition with middle-chain triglycerides (MCT), which led to a slight reduction of the drained volume of about 500 ml daily during the first week. To enable outpatient monitoring and to wait for a delayed treatment response, a PleurX^®^ (Denver Biomedical Inc., CO, USA) catheter was implanted and the patient was planned for regular controls. Although the overall drained volume significantly decreased during the following weeks, our patient reported a weight loss of about 6 kg, persistence of peripheral lymphedema, and nail discoloration, despite treatment adherence. To evaluate further treatment options, lymphangiography was conducted, which showed anomalous lymphatic drainage with chylolymphatic reflux into mediastinal lymphatic vessels (Figs. 7, 8). On the following day, percutaneous lymphatic embolization with lipidol was performed through an inguinal access site. Subsequent radiographic assessment including CT thorax indicated promising results with no signs of postinterventional complications and a reduction of the drained volume to about 100 ml daily during the following week. Lymphedema was treated with compression bandages, manual drainage, and a mechanical decongestive device. To support nail regeneration, our patient was prescribed treatment with topical tocopherol (vitamin E) and oral zinc supplementation.

**Table 1:** Laboratory assessment

Parameters	Pleural fluid	Serum
Protein (g/dl)	4.43	6.4
LDH (U/l)	124	144
Glucose (mg/dl)	103	78
pH	7.40	7.42
Triglycerides (mg/dl)	127	83
Cholesterol (mg/dl)	98	218
LDL (mg/dl)	79	130
HDL (mg/dl)	23	71

Results from laboratory assessment of pleural fluid and serum. *LDH* lactate dehydrogenase, *LDL* low-density lipoprotein, *HDL* high-density lipoprotein

## Discussion

The diagnosis of YNS is based on the characteristic clinical symptoms and after exclusion of other more common diseases. The diagnostic workup of our patient followed a step-wise approach beginning with the ultrasound-guided puncture of the pleural effusion, whose further laboratory assessment confirmed the presence of a chylothorax. From a pathophysiological perspective, a chylothorax can be due to trauma, e.g. following surgical interventions, or is caused by a primary tumorous or inflammatory disease [6]. The common underlying condition is an impaired lymphatic drainage, which leads to an accumulation of chyle into the pleural space. In most cases, a chylous pleural effusion presents as an exsudate with elevated pleural LDH and protein levels on laboratory analysis. Protein loss can be explained by either an obstruction or an increased permeability of intrapleural lymphatic vessels [7]. On the basis of the findings by Staats *et al.* (1980), a pleural effusion triglyceride level > 110 mg/dl combined with a cholesterol concentration < 200 mg/dl confirmed the diagnosis of a chylothorax [8]. Anatomically, a predominantly left-sided pleural effusion indicates that the lesion of the thoracic duct is located above the fifth thoracic vertebra, whereas a more caudal damage usually causes leakage into the right pleural cavity [9].

Regarding our patient, the observed mediastinal lipoma located on the level of the third and fourth thoracic vertebrae could have obstructed the drainage of the thoracic duct into the left subclavian vein. This would explain the chylous reflux into mediastinal lymphatic collaterals on CT lymphangiography. Since there were no signs of compression of other mediastinal structures, and presuming an increased intraoperative risk, an interventional embolization of the thoracic duct was preferred over surgical tumor resection. Percutaneous lymphatic embolization carries a lower procedural risk with a similar clinical outcome compared with surgical ligature of the thoracic duct [10]. The interventional approach was chosen after conservative management with intravenous MCT and subsequent fat-restrictive diet did not lead to an improvement of symptoms. Nutritional approaches generally intend to reduce the overall lymphatic drainage, thereby enabling the spontaneous closure of the leakage in the thoracic duct.

The clinical diagnosis of lymphedema was based on the patient’s history and the physical examination. Our patient reported to have noticed the onset of progressive swelling of his distal extremities about 3 years ago. Consistent with lymphedema, we noticed nonpitting, circumferential edema of the lower extremities, which had been resistant to diuretic treatment. A positive Stemmer sign, which is the inability to pinch the dorsal side of the patient’s second toe, substantiated the clinical diagnosis [11]. Anomalous lymphatic vessels with collaterals demonstrating chylolymphatic reflux were observed on CT lymphangiography as the structural correlate of our patient’s symptoms. Treatment-wise, flexible bandages, manual drainage, and decongestive therapy with a mechanical device were prescribed. Although these treatments are symptomatic and intend to prevent the need for surgical interventions, successful implementation of physical therapy has been related to an improvement of nail changes [12].

Regarding nail symptoms, the diagnosis can be made after exclusion of a mycosis- or substance-related cause of the discoloration. While some authors discuss an initially impaired lymphatic drainage as the cause of nail symptoms, others suggest a secondary lymphatic obstruction due to stromal fibrosis as the underlying pathophysiological mechanism [13]. Although microscopical findings in YNS are numerous, none of them are specific enough to make the diagnosis. The yellow discoloration of nails, which is observed in many hereditary or acquired pathologies of the lymphatic system, could be explained by an accumulation of lipofuscin pigments [14]. In contrast to other lymphatic disorders, nail symptoms in YNS involve a thickening due to increased horizontal and decreased longitudinal growth. Correspondingly, a replacement of the subungual stroma by a more dense, fibrous tissue can be observed on light microscopy. Treatment of nail changes includes topical therapy with antimycotics of the azole group and terbinafine, which are, in addition, associated with a stimulatory effect on longitudinal nail growth [15]. Treatment with topical or oral vitamin E can lead to a reduction of accumulated lipofuscin [16]. Zinc deficiency is related to different nail pathologies, and its supplementation is described in cases of successfully treated YNS [17]. Regarding our patient, we decided for a local treatment with 5% vitamin E solution and 30 mg zinc once daily.

## Conclusion

Our patient presented with all three characteristic features of YNS, which could be explained by anomalous lymphatic drainage presumably linked to a paratracheal lipoma compressing the thoracic duct. An interventional approach with percutaneous lymphatic embolization was chosen after conservative treatment had not caused a significant improvement of the initial symptoms. The complexity of the underlying pathophysiology in YNS and the lack of treatment guidelines demonstrate the necessity for more research on this rare disorder.

## Data Availability

Not applicable.

## Abbreviations

CT: Computed tomography
FEV1: Forced expiratory volume
FVC: Forced vital capacity
H&E: Hematoxylin and eosin
HDL: High density lipoprotein
LDH: Lactate dehydrogenase
LDL: Low-density lipoprotein
MCT: Middle-chain triglycerides
PAS: Periodic acid–Schiff stain
YNS: Yellow nail syndrome
